# Immunoarchitectural characterization of a human skin model reconstructed *in vitro*

**DOI:** 10.1590/S1516-31802009000100007

**Published:** 2009-05-15

**Authors:** Luís Ricardo Martinhão Souto, José Vassallo, Jussara Rehder, Glauce Aparecida Pinto, Maria Beatriz Puzzi

**Affiliations:** 1 MD, MSc. Postgraduate (PhD) student of Surgery, School of Medical Sciences, Universidade Estadual de Campinas (Unicamp), Campinas, São Paulo, Brazil.; 2 MD, PhD. Titular professor, Department of Pathological Anatomy, School of Medical Sciences, Universidade Estadual de Campinas (Unicamp), Campinas, São Paulo, Brazil.; 3 BSc. Chief biologist, Laboratory of Molecular Biology and Skin Cell Culture, School of Medical Sciences, Universidade Estadual de Campinas (Unicamp), Campinas, São Paulo, Brazil.; 4 BSc, PhD. Biomedical researcher, Laboratory of Experimental Pathology, Women’s Full Healthcare Center, Universidade Estadual de Campinas (Unicamp), Campinas, São Paulo, Brazil.; 5 MD, PhD. Associate professor of Dermatology, Department of Internal Medicine. Head of the Skin Cell Culture Laboratory, School of Medical Sciences, Universidade Estadual de Campinas (Unicamp), Campinas, São Paulo, Brazil.

**Keywords:** Burns, Cytokeratin, Immunohistochemistry, Skin transplantation, Tissue engineering., Queimaduras, Citoqueratina, Imunoistoquímica, Transplante de pele, Engenharia tissular.

## Abstract

**CONTEXT AND OBJECTIVE:**

Over the last few years, different models for human skin equivalent reconstructed *in vitro* (HSERIV) have been reported for clinical usage and applications in research for the pharmaceutical industry. Before release for routine use as human skin replacements, HSERIV models need to be tested regarding their similarity with *in vivo* skin, using morphological (architectural) and immunohistochemical (functional) analyses. A model for HSERIV has been developed in our hospital, and our aim here was to further characterize its immunoarchitectural features by comparing them with human skin, before it can be tested for clinical use, e.g. for severe burns or wounds, whenever ancillary methods are not indicated.

**DESIGN AND SETTING:**

Experimental laboratory study, in the Skin Cell Culture Laboratory, School of Medical Sciences, Universidade Estadual de Campinas.

**METHODS:**

Histological sections were stained with hematoxylin-eosin, Masson’s trichrome for collagen fibers, periodic acid-Schiff reagent for basement membrane and glycogen, Weigert-Van Gieson for elastic fibers and Fontana-Masson for melanocytes. Immunohistochemistry was used to localize cytokeratins (broad spectrum of molecular weight, AE1/AE3), high molecular weight cytokeratins (34bE12), low molecular weight cytokeratins (35bH11), cytokeratins 7 and 20, vimentin, S-100 protein (for melanocytic and dendritic cells), CD68 (KP1, histiocytes) and CD34 (QBend, endothelium).

**RESULTS:**

Histology revealed satisfactory similarity between HSERIV and *in vivo* skin. Immunohistochemical analysis on HSERIV demonstrated that the marker pattern was similar to what is generally present in human skin *in vivo*.

**CONCLUSION:**

HSERIV is morphologically and functionally compatible with human skin observed *in vivo*.

## RESUMO

**CONTEXTO E OBJETIVO:**

Nos últimos anos, diferentes modelos de pele humana reconstruída *in vitro* (PHRIV) foram descritos para uso clínico e aplicações em pesquisa na indústria farmacêutica. Antes de serem liberados para uso rotineiro como substitutos de pele humana, os modelos de PHRIV necessitam de testes (estudos) comparativos com a pele humana *in vivo*, por meio de análises morfológica (arquitetural) e imunoistoquímica (funcional). O objetivo deste trabalho é estudar as características imunoistoquímicas de um modelo de PHRIV desenvolvido em nosso serviço, comparando-as com a pele humana, para que esse modelo de PHRIV possa vir a ser testado clinicamente em casos de queimaduras e ulcerações de pele nos quais métodos tradicionais de tratamento não estejam indicados.

**TIPO DE ESTUDO E LOCAL:**

Estudo experimental laboratorial realizado no Laboratório de Cultura de Células da Pele da Faculdade de Ciências Médicas da Universidade Estadual de Campinas (FCM/Unicamp), Campinas, São Paulo, Brasil.

**MÉTODOS:**

Cortes histológicos foram corados com hematoxilina-eosina, tricrômio de Masson para fibras colágenas, ácido periódico-reagente de Schiff para membrana basal e glicogênio, Weigert-Van Gieson para fibras elásticas e Fontana-Masson para melanócitos. Estudo imunoistoquímico foi realizado para identificar citoqueratinas de amplo espectro de pesos moleculares (AE1/AE3), citoqueratinas de alto peso molecular (34bE12), citoqueratinas de baixo peso molecular (35bH11), citoqueratinas 7 e 20, vimentina, proteína S-100 (para melanócitos e células dendríticas), CD68 (KP1, histiócitos) e CD34 (QBend, endotélio).

**RESULTADOS:**

A histologia revelou similaridade satisfatória entre PHRIV e a pele *in vivo*. O estudo imunoistoquímico da PHRIV demonstrou padrão semelhante de marcadores usualmente presentes na pele humana *in vivo*.

**CONCLUSÃO:**

A PHRIV estudada é morfológica e funcionalmente compatível com a pele humana observada *in vivo*.

## INTRODUCTION

Over the last few years, different models for human skin equivalent reconstructed *in vitro* (HSERIV) have been developed, containing associations between dermis (or a dermal equivalent) and epidermis.^1^^,^^2^^,^^3^^,^^4^^,^^5^^,^^6^ These models represent an alternative system for testing pharmacological products in the place of laboratory animals.^7^ The European Union, through its “Sixth Amendment to the European Community Cosmetic Directive”, has been aiming towards banning the use of animals in tests for certain types of cosmetic ingredients, combinations of ingredients and final products since January 1, 1998.^8^ Furthermore, the skin substitutes obtained by tissue engineering nowadays represent an advanced therapeutic option for treating cutaneous lesions.^9^

The most recent approach, offering the best prospects for obtaining complex tissues in laboratories, is the use of biodegradable matrices as “models” to direct cell growth. These matrices are normally filled with live cells, derived from biopsies or from multipotent cells, that proliferate, organize and produce cellular and extracellular matrices. During the formation of cellular and extracellular matrices by the cells, the initially existing matrices are degraded, absorbed or metabolized.^10^

The following prerequisites are needed for a human skin equivalent to be used for scientific purposes:

- Formation of a corneous stratus *in vitro* in the epidermis, thereby forming a mechanical barrier similar to the one found *in vivo*;
- Production of the intracellular lamellar glycogen corpuscles that are present in the granular layer of the epidermis;
- Lamellar (cuticular) appearance for the epidermis.^11^

After meeting the scientific criteria and before the human skin equivalent can be used for studies or for clinical treatment in humans, it needs to be considered similar to human skin *in vivo*. One of the ways to obtain approval for the use of an *in vitro* human skin equivalent in people is to perform prior tests on animals. However, there is longstanding concern^12^ about not using animals for laboratory tests and, as mentioned above, there are now moral and legal barriers against this.^11^ Another efficient method that does not cause moral or legal conflicts is to perform studies at the molecular and histochemical level on the human skin equivalents obtained.

Immunohistochemistry is a simple and widely used method for detecting a number of cellular and extracellular antigens, either in fresh tissues and cells or in fixed paraffin-embedded specimens. These antigens mostly correspond to protean filaments that are present in the cells, which may allow better understanding of the physiopathology of normal and diseased skin.^13^

In a previous study by our group, a specific model for HSERIV was reported.^6^ The resulting tissue consisted of an association of dermis and epidermis that was morphologically compatible with human skin *in vivo*. Our objective here was to functionally validate this skin model,^6^ through analysis of the tissue architecture and expression of specific protean markers of differentiation, with the aim of achieving acceptance for this human skin model for future use in clinical studies.

## METHODS

This study was performed at the Skin Cell Culture Laboratory of the School of Medical Sciences, Universidade Estadual de Campinas (Unicamp), Campinas, São Paulo, Brazil. The technique used for obtaining reconstructed human skin *in vitro* was the one described by Souto et al.^6^ and it is summarized here.

After obtaining skin samples from patients who had undergone mammoplasty or abdominoplasty, cell cultures were performed in specific growth media for each cell type:

- Culturing of epidermal epithelial cells in a culture medium for keratinocytes (Gibco BRL, Grand Island, New York, United States, catalog number 10785-012);
- Culturing of melanocytes in a specific culture medium (MCDB 153; Sigma Chemical Co., St. Louis, Missouri, United States, code M-7403);
- Culturing of fibroblasts in M199 culture medium (Gibco, catalog number 31100-035).

All the culture media were supplemented with L-glutamine (2 mM/ml), penicillin (100 IU/ml), streptomycin (0.1 mg/ml) (Gibco BRL, Grand Island, New York, United States, catalog number 10378-016) and 10% bovine fetal serum (Gibco, catalog number 10437-028). Epidermal growth factor (5 hg/ml) (Gibco, catalog number 10450-013), bovine pituitary extract (50 μg/ml) (Gibco, catalog number 13028-014), hydrocortisone (0.6 μg/ml) (Sigma, code H-0888) and bovine insulin (3 μg/ml) (Gibco, catalog number 13007-018) were also added to the culture medium for keratinocytes.

After obtaining specific cultures for each cell type, fibroblasts were injected using a syringe, into a permeable and porous acellular mold (matrix) of bovine collagen type I, in block form (Gen-col, block collagen, code 961.25, Genius line, Baumer Biomaterials Division, Mogi Mirim, São Paulo, Brazil), measuring approximately 10 x 15 x 25 millimeters. The volume injected was 2.0 x 10^6^ cells (fibroblasts), in M199 culture medium (Gibco, catalog number 31100-035), supplemented with L-glutamine (2 mM/ml), penicillin (100 IU/ml), streptomycin (0.1 mg/ml) (Gibco, catalog number 10378-0160) and 10% bovine fetal serum (Gibco, catalog number 10437-028).

The collagen type I matrix containing fibroblasts was placed on a Petri dish of about 10.0 cm^2^ and immersed in a culture medium for fibroblasts (M199; Gibco, catalog number 31100-035), supplemented with L-glutamine (2 mM/ml), penicillin (100 IU/ml), streptomycin (0.1 mg/ml) (Gibco, catalog number 10378-016) and 10% bovine fetal serum (Gibco, catalog number 10437-028) and placed in an incubator at 37 °C, with CO_2_ tension of 5%.

The culture medium was changed three times a week. Reconstructed human dermis *in vitro* could be obtained in seven days. Epidermal cells (ratio between melanocytes and keratinocytes of 1:4) were grown on this human dermis reconstructed *in vitro* on the Petri dish, mixed with 5.0 x 10^6^ cells with about 1.0 x 10^6^ melanocytes and 4.0 x 10^6^ keratinocytes. The system was immersed in a culture medium for skin, formed by three parts of IMDM (Iscove’s Modified Dulbecco’s Medium; Gibco, catalog number 12200-036) plus one part of culture medium for keratinocytes (Defined Keratinocyte Medium; Gibco, catalog number 10785-012), 10% bovine fetal serum (Gibco, catalog number 10437-028) and supplements of L-glutamine (2 mM/ml), penicillin (100 IU/ml), streptomycin (0.1 mg/ml) (Gibco, catalog number 10378-016) and 1.5 mM Ca^2+^.

The culture medium was changed three times a week. HSERIV containing dermis and epidermis could be obtained in seven days (from the “ready” human dermis reconstructed *in vitro*). After obtaining the HSERIV composed of associated dermis and epidermis, it was subjected to architectural (morphological) and immunohistochemical (functional) analysis.

The HSERIV was fixed in 10% formalin and embedded in paraffin. Histological sections of 4 μm in thickness were stained with hematoxylin-eosin (HE), Weigert-Van Gieson for elastic fibers, periodic acid-Schiff (PAS) to delineate the basement membrane and Fontana-Masson silver impregnation to detect melanin pigmentation.

Immunohistochemical tests were performed using the following antibodies: anti-pan cytokeratin (AE1/AE3, code M3515; diluted at 1:100), high molecular weight cytokeratins (34bE12, code M0630; diluted at 1:50), low molecular weight cytokeratins (35bH11, code M0631; diluted at 1:50), cytokeratin 7 (code M7018; diluted at 1:50), cytokeratin 20 (code M7019; diluted at 1:50), vimentin (clone V9, code M0725; diluted at 1:100), S-100 protein (polyclonal, code Z0311; diluted at 1:1000), anti-CD68 (clone KP1, code M0814; diluted at 1:100; histiocytic lineage) and anti-CD34 (code M7165; diluted at 1:50; endothelial cells). All markers were obtained from DakoCytomation (Dako), Carpenteria, California, United States. All antibodies were diluted in 1% bovine serum albumin (BSA) in phosphate saline buffer, at pH 7.6.

After deparaffination and hydration of the histological sections, they were then subjected to blocking of endogenous peroxidase, and antigen recovery in a steamer (T-Fall, Dijon, France) with 10 mM citrate buffer at pH 6.0, at 90 °C for 30 minutes. The slides were incubated with the primary antibodies described above for 18 hours (overnight) at 4 °C. The antigen-antibody binding was detected by means of the EnVision peroxidase system (code K1491; Dako). Staining was achieved using 3,3’-diaminobenzidine (code D-5637; Sigma, St. Louis, Missouri, United States) diluted in 3% H_2_O_2_ in phosphate saline buffer (PBS, pH 7.6). The positive controls consisted of skin fragments containing nevocellular nevi. The same specimens were used as negative controls, by omitting the primary antibody and replacing it with incubation with PBS buffer. Positive reactivity was shown by brown staining in the cytoplasm, as seen under an optical microscope.

The entire experiment was successfully performed in triplicate, in order to validate the analysis.

This investigation was approved by and conducted in accordance with the statements of the Research Ethics Committee of the School of Medical Sciences, Universidade Estadual de Campinas (Unicamp) and it complied with the Declaration of Helsinki, 1975, as amended in 1983.

## RESULTS

The histological features of HSERIV have already been described elsewhere.^6^ Weigert-Van Gieson staining showed a small number of thin elastic fibers in the dermal equivalent. The PAS reaction delineated the basement membrane as a continuous thin layer between the epidermis and dermis. Fontana-Masson silver impregnation showed melanin pigmentation in scattered cells located in the basal layer, just above the basement membrane.

**Figure 1.**
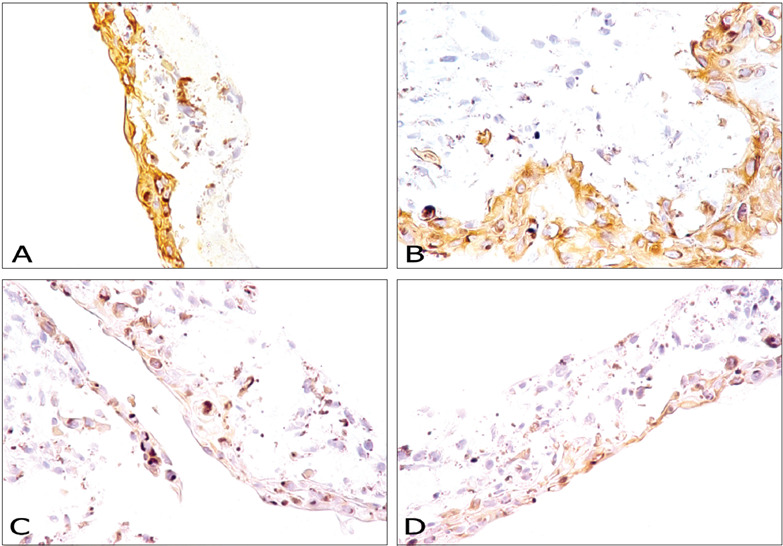
Immunohistochemical characterization of a human skin model reconstructed *in vitro* based on analysis of the cytokeratins present in epithelial cells. (A) Human skin reconstructed *in vitro*. Immunohistochemical analysis on anti-cytokeratin antibodies with a broad spectrum of molecular weights (AE1/AE3). Strong positive appearance in the epidermis (original magnifi cation 400 X). (B) Human skin reconstructed *in vitro*. Immunohistochemical analysis on anti-cytokeratin antibodies of high molecular weight (34βE12). Strong positive appearance in the epidermis (original magnifi cation 400 X). (C) Human skin reconstructed *in vitro*. Immunohistochemical analysis on anti-cytokeratin antibodies of low molecular weight (35βH11). Moderate to weak positive appearance in the epidermis (original magnifi cation 400 X). (D) Human skin reconstructed *in vitro*. Immunohistochemical analysis on anti- cytokeratin antibody 7. Weak positive apperance in the epidermis (original magnifi cation 400 X).

The immunohistochemical analysis on HSERIV showed that all the epithelial cells in all layers were strongly positive for pan cytokeratin AE1/AE3 and for high molecular weight cytokeratins (34bE12), as described in normal epidermis. Scattered epithelial cells presented lower intensity of reaction to low molecular weight cytokeratins (35bH11) and cytokeratins 7 and 20 (Figure 1).

**Figure 2.**
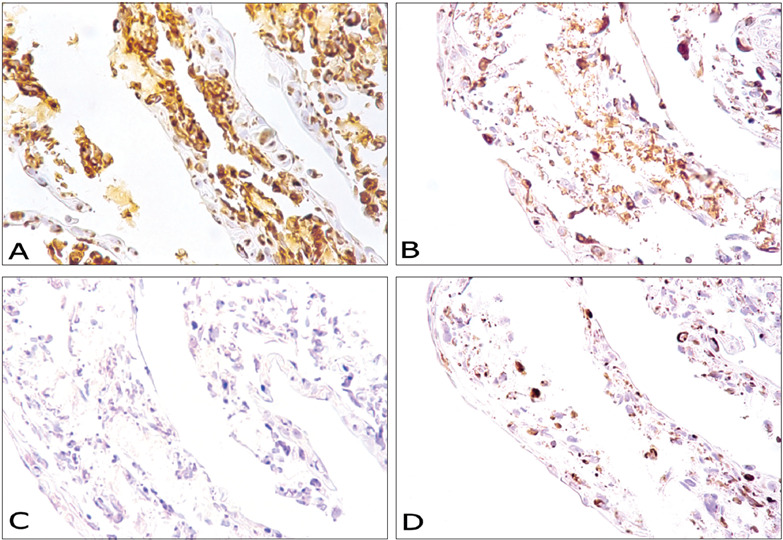
Immunohistochemical characterization of a human skin model reconstructed *in vitro* based on analysis of mesenchymal cells. (A) Human skin reconstructed *in vitro*. Immunohistochemical analysis on anti-vimentin antibodies. Strong positive appearance in the dermis (original magnifi cation 400 X). (B) Human skin reconstructed *in vitro*. Immunohistochemical analysis on anti-S-100 protein antibodies. Weak to moderate positive appearance in the epidermis (original magnifi cation 400 X). (C) Human skin reconstructed *in vitro*. Immunohistochemical analysis on negative anti-CD68 antibodies (original magnifi cation 400 X). (D) Human skin reconstructed *in vitro*. Immunohistochemical analysis on negative anti-CD34 antibodies (original magnifi cation 400 X).

Mesenchymal markers were recognized in the underlying tissue: all dermal cells were strongly positive for vimentin, while a few scattered melanocytic cells stained for S100 protein. On the other hand, no histiocytic or endothelial cells were found, as demonstrated with antibodies for CD68 and CD34, respectively (Figure 2).

## DISCUSSION

The stratification of the epidermis in the HSERIV presented characteristics similar to what is found *in vivo*, although no formation of a real *stratum spinosum* was seen, and therefore the *stratum corneum* was extremely thin. The lack of formation of a real *stratum spinosum* in the epidermis of HSERIV models had already been reported in previous studies.^14^ The formation of a thin pellicle where the *stratum corneum* should be located might be due to the short period of time for which the newly formed epidermis remained exposed to the air-liquid interface, as discussed by others.^15^

With regard to the extracellular matrix observed in the model that we developed,^6^ it seems possible that the bovine collagen was initially used by the human fibroblasts as a source of nutrition and also to regulate and orientate their growth. In a second stage, bovine collagen would be replaced by collagen from the injected fibroblasts, as accepted in other studies.^1^^,^^4^ The presence of only a few elastic fibers in the skin model, compared with normal skin (Weigert-Van Gieson staining), was probably due to the short time for which the cells remained in the culture medium, as mentioned earlier.^15^

The presence of a thin basement membrane in HSERIV (as seen via the PAS technique) indirectly demonstrates the formation of a completely differentiated skin equivalent. As already stated, interactions between the epidermis and dermis, which are dependent on keratinocytes and dermal fibroblasts, are important for the production of laminin and collagen type IV (components of the basement membrane).^16^ In addition, the pigmentation detected by Fontana-Masson silver impregnation corresponds to scattered melanin-producing cells, next to the basement membrane.

The use of immunohistochemical techniques to classify cell histogenesis is becoming more and more important, as the generation and application of skin equivalents grown *in vitro* are increasingly frequently achieved.^17^ These techniques are important for determining whether the cells present in skin equivalents *in vitro* correspond to the immune profile of cells from normal skin *in vivo*, before these equivalents can be used in research or therapeutics.^18^ A panel of immunomarkers is needed for better characterization of different proteins of the epithelium and mesenchyma, since they may differ according to function and the cell differentiation and maturation status.^13^

Up to now, 20 types of cytokeratins have been identified‚^19^ with important functions as members of the cytoskeleton.^13^ The cytokeratins expressed in normal skin are of types 5 and 14 (basal layer of the epidermis), 1 and 10 (*stratum spinosum*) and 2 and 11 (*stratum granulosum*). In cases in which there is keratinocyte proliferation (e.g. psoriasis or healing), the cells of the suprabasal layer synthesize keratins 6 and 16.^19^^,^^20^

The AE1 antibody reacts with cytokeratins 10, 15, 16 and 19, and AE3 with cytokeratins 1, 2, 3, 4, 5, 6 and 8. A cocktail of both of these antibodies has been developed in order to react with a wide range of cytokeratins: the pan cytokeratin AE1/AE3. The antibody to high molecular weight cytokeratins (34bE12) reacts with cytokeratins 1, 5, 10 and 14, and the antibody to low molecular cytokeratins (35bH11) reacts with cytokeratins 8 and 18.^19^^,^^21^ Detection of cytokeratin 35bH11 is useful in determining the glandular nature of some tumors, such as adenocarcinoma and Paget’s disease.^13^ Cytokeratin 20 (CK20) is expressed in the gastric epithelium, urothelium, intestinal epithelium and Merkel cells of the skin.^13^ Detection of cytokeratin 7 is mainly achieved in pulmonary, mammary, ovarian, endometrial and neuroendocrine tissues. In the skin, cytokeratin 7 may be expressed in basal cell carcinomas and trichoepitheliomas.^22^ A given type of epithelium or epithelial cell can be characterized by the specificity of the cytokeratins expressed, and the cytokeratins produced by cultured cells (*in vitro*) are usually very similar to the cytokeratins expressed in normal tissues and in tumors.^23^ In the present skin model, the most intense and extensive staining was achieved with AE1/AE3 and 34bE12, as is usually the case in normal skin. The presence of scattered cells that were less intensely positive for 35bH11 and cytokeratins 7 and 20 may reflect immaturity of some of the cells at the development stage at which they were studied.^21^^,^^23^

Melanocytes express S-100 protein, which was originally detected in glial cells.^13^ The polyclonal antibody to S-100 protein is routinely used to detect normal and altered melanocytes (melanomas), due to its high sensitivity.^17^ However, in the skin, antibodies to S-100 protein are not specific for melanocytes, since they can also identify Langerhans and Schwann cells,^17^^,^^24^ sensory corpuscles and sudoriparous gland cells.^24^ In our skin model, the topography of S-100-positive cells among basal cells, together with the detection of pigment by Fontana-Masson silver impregnation is consistent with their melanocytic origin.

In the skin, CD68 is present on the surface of macrophages and in Langerhans cells, while in other types of tissue, it is located in monocytes and macrophages.^25^ The human progenitor cell antigen CD34 is a surface antigen expressed by normal hematopoietic progenitors of the bone marrow. In normal skin, the expression of CD34 can be observed in perivascular interstitial cells of the connective adventitial tissue and in the endothelium of blood vessels.^13^^,^^26^ The absence of CD68 and CD34 in our HSERIV was expected, since neither macrophages nor hematopoietic progenitor cells were added during the process of skin reconstruction *in vitro*. In addition, the skin equivalent described here did not contain blood vessels that could have been positive for CD34.

## CONCLUSION

There is a clear similarity between the HSERIV presented here and human skin *in vivo.* Neither abnormalities nor evident degenerative alterations were seen in the cells, as demonstrated by morphological and immunohistochemical analyses. Our HSERIV may be considered to be similar to human skin *in vivo* and appropriate for laboratory/pharmaceutical or clinical and therapeutic studies. We believe that it may represent a promising substitute in cases of burns and chronic skin ulcers, with the aim of solving the problem of the need for large amounts of skin for transplantation in patients with scarce donor sites.
